# A Descriptive Analysis of the Use of Chemical, Biological, Radiological, and Nuclear Weapons by Violent Non-State Actors and the Modern-Day Environment of Threat

**DOI:** 10.1017/S1049023X23000481

**Published:** 2023-06

**Authors:** Derrick Tin, Lenard Cheng, Heejun Shin, Ryan Hata, Fredrik Granholm, George Braitberg, Gregory Ciottone

**Affiliations:** 1. Disaster Medicine Fellowship; Department of Emergency Medicine, Beth Israel Deaconess Medical Center and Harvard Medical School, Boston, Massachusetts USA; 2. Department of Critical Care Medicine, University of Melbourne, Parkville Victoria, Australia; 3. Swedish Air Ambulance (SLA), Mora, Sweden; Adjunct Faculty, BIDMC Disaster Medicine Fellowship, Beth Israel Deaconess Medical Center, Boston, Massachusetts USA

**Keywords:** CBRNe, Counter-Terrorism Medicine, disaster medicine, weapons of mass destruction

## Abstract

**Introduction:**

The use of chemical, biological, radiation, and nuclear (CBRN) weapons is not new, and though rare, it is an issue of concern around the world due to their ability to cause large-scale mass-casualty events and their potential threat to global stability. The purpose of this study is to explore the use of CBRN weapons by non-state actors through analysis of the Violent Non-State Actor (VNSA) CBRN Event database, and aims to better inform health care systems of the potential risks and consequences of such events.

**Methods:**

Data collection was performed using a retrospective database search through the VNSA CBRN Event database.

**Results:**

A total of 565 events were recorded. Five hundred and five (505) events (89.4%) involved single agents while 60 events (10.6%) involved multiple agents. Fatalities numbered 965 for chemical agents, 19 for biological agents, and none for radiological and nuclear events. Injuries numbered 7,540 for chemical agents, 59 for biological agents, 50 for radiological events, and none for nuclear attacks. Fatality and injury per attack was 2.22 and 17.37, respectively, for chemical event agents and 0.15 and 0.48, respectively, for biological event agents.

**Conclusion:**

Violent Non-State Actors were responsible for 565 unique events around the world involving the use of CBRN weapons from 1990-2020. The United States (118), Russia (49), and Iraq (43) accounted for the top three countries where these events occurred. While CBRN events remain relatively rare, technological advances have the potential to facilitate the use of such weapons as part of a hybrid warfare strategy with significant repercussions for civilian health and health care systems.

## Introduction

The use of chemical, biological, radiation, and nuclear (CBRN) weapons is not new, and though rare, it is an issue of concern around the world due to their ability to cause large-scale mass-casualty events and their potential threat to global stability. While there exists a number of internationally ratified conventions governing or banning the use of such weapons by state actors, concerns about non-state actors acquiring or developing such weapons remain. Defined by the United Nations (UN) Security Council (New York USA) as “individuals or entities not acting under the lawful authority of any State,” non-state actors acquiring and using CBRN or related weapons have been signaled by the UN Office of Drugs and Crime (Vienna, Austria) as “one of the gravest concerns of our time.”^1,2^

Deadly chemicals are readily available in the industrial sector and home-made bioweapons are increasingly plausible as biotechnologies advance.^3–5^ In addition, the threat of artificial intelligence (AI) looms as a tool to create innovative chemical weapons that might not otherwise have been conceived of.^6^ Further, radiation sources used commonly in the medical sector are vulnerable to theft and modifications and then used to create dirty bombs, and nuclear plants are vulnerable to both cyber hacks and physical damage, which could lead to meltdowns and wide-spread human, structural, and environmental devastation.^7–9^

The purpose of this study is to explore the use of CBRN weapons by non-state actors through analysis of the Violent Non-State Actor (VNSA) CBRN Event database, and aims to better inform health care systems of the potential risks and consequences of such events.

## Methods

Data collection was performed using a retrospective database search through the VNSA CBRN Event database.^10^ The database was developed and is maintained by the Unconventional Weapons & Technology Division of the National Consortium for the Study of Terrorism and Responses to Terrorism (START; College Park, Maryland USA) and compiles data solely and exclusively from publicly accessible open-source materials, and does not contain any personal identifying information. The latest update of the database at the time of writing in December 2022 recorded events ranging from 1990 through 2020. Only events meeting all three criteria as set out by the VNSA CBRN Event database are included:

- Criteria 1. The event must be intentional rather than inadvertent. The event must result from a conscious calculation on the part of a threat actor/alleged threat actor.
- Criteria 2. The event must entail some level of violence, planned violence, or threat of violence, including property violence.
- Criteria 3. The threat actor/alleged threat actor must be an individual or group operating independently from the state. Threat actors receiving support in the form of materials or training from a state may still be included, provided the state is not exercising direct operational control over the threat actor’s/alleged threat actor’s planning or operations.

Events committed by state actors or those with a purely criminal nature and no ideological motives are excluded from the database.

The VNSA CBRN Event database was downloaded in Excel (Version 16.71; Microsoft Corp.; Redmond, Washington USA) format. All available fields were examined, and the fields most relevant to the research objective were reported: event identification number, year, location, event agent, total fatalities, and injuries.

The event agent fields extracted from the database were classified into chemical, biological, toxin, radiological, and nuclear. Events involving multiple agents were classified into multiple respective categories; for example, a single event employing both chemical and biological agents contributed a count each under chemical and biological event agents. Due to contention over whether toxins are biological or chemical agents, the database allocated a classification “toxin” which is further sub-classified as either primarily biological or primarily chemical. For the purpose of this study, primarily biological toxin events were counted as biological events and primarily chemical toxin events were counted as chemical events.

Specific event agents were coded by the database authors according to the intended rather than the eventual agents or weapons. For example, if the actor intended to use hydrogen cyanide but eventually acquired potassium cyanide, hydrogen cyanide was coded as the agent for the event.

Results were exported into a separate Excel spreadsheet for analysis and confirmed by two independent data extractors.

## Results

**Figure 1.**
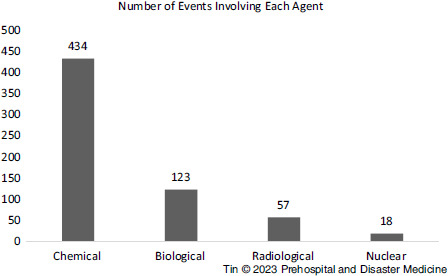
Number of Events Involving Chemical, Biological, Radiological, and Nuclear Agents.

**Figure 2.**
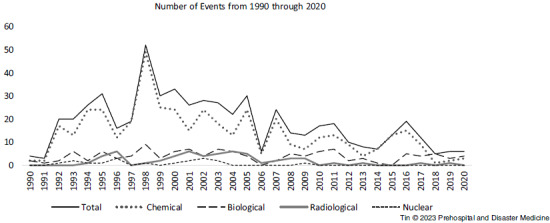
Total and Agent-Specific Number of Events from 1990 through 2020.

A total of 565 events were recorded. Five hundred and five (505) events (89.4%) involved single agents while 60 events (10.6%) involved multiple agents, elaborated in Table 1. Three hundred thirty-four (434) events involved chemical agents, 123 involved biological agents, 57 involved radiological agents, and 18 involved nuclear agents (Figure 1). Total and agent-specific number of events from 1990 through 2020 are represented in Figure 2. To clarify, these numbers reflect the number of times each agent was involved, including events with multiple agents, and hence exceed the total number of events (ie, a single event involving two agents will be counted twice in this figure).

**Table 1.** Number of Events Involving Chemical, Biological, Radiological, or Nuclear Agents and Combinations in Events Involving Mixed Agents

Event Agent	Number of Events
Chemical Only	380 (67.3%)
Biological Only	74 (13.1%)
Radiological Only	40 (7.1%)
Nuclear Only	11 (2.0%)
C+B+R+N	1 (0.2%)
C+B+R	4 (0.7%)
C+B+N	1 (0.2%)
C+R+N	1 (0.2%)
C+B	42 (7.4%)
C+R	5 (0.9%)
B+R	1 (0.2%)
R+N	5 (0.9%)

Abbreviations: C, chemical; B, biological; R, radiological; N, nuclear.

The ten most common chemical agents were unknown poison (n = 83; 19.1%), unknown chemical (n = 74; 17.1%), hydrogen cyanide (n = 47; 10.8%), chlorine (n = 43; 9.9%), butyric acid (n = 26; 6.0%), sodium cyanide (n = 23; 5.3%), sarin (n = 20; 4.6%), mustard gas (n = 18; 4.1%), VX nerve agent (n = 14; 3.2%), and unknown cyanide (n = 14; 3.2%). The five most common biological agents were ricin (n = 51; 41.5%), unknown biological (n = 32; 26.0%), bacillus anthracis (n = 18; 14.6%), clostridium botulinum toxin (n = 13; 10.6%), sewage (n = 4; 3.3%), and human immunodeficiency virus (n = 3; 2.4%).

Total fatalities and injuries numbered 980 and 7,649, respectively. Fatalities numbered 965 for chemical agents, 19 for biological agents, and none for radiological and nuclear events. Injuries numbered 7,540 for chemical agents, 59 for biological agents, 50 for radiological events, and none for nuclear attacks. Fatality and injury per attack was 2.22 and 17.37, respectively, for chemical event agents and 0.15 and 0.48, respectively, for biological event agents (Table 2).

**Table 2.** Fatalities and Injuries of Events Classified According to Event Agent

Type	Total Fatalities	Total Injuries	Fatalities per Event	Injuries per Event
Chemical	965	7,540	2.22	17.37
Biological	19	59	0.15	0.48
Radiological	0	50	NA	0.88
Nuclear	0	0	NA	NA

Out of the 565 total events, 386 events (68.3%) took place in ten countries listed in Table 3, while the remaining 179 events (31.7%) took place in 55 countries. Specifically, the ten countries registering the greatest number of events were the United States (n = 118; 20.9%), Russia (n = 49; 8.7%), Iraq (n = 43; 7.6%), Japan (n = 40; 7.1%), United Kingdom (n = 30; 5.3%), Afghanistan (n = 25; 4.4%), China (n = 24; 4.3%), Israel (n = 22; 3.9%), Cambodia (n = 22; 3.4%), and India (n = 16; 2.8%). These ten countries registered 537 (54.8%) fatalities and 6,612 (86.4%) injuries. Fatality per attack was highest at 12.0 in Cambodia, and injury per attack was highest in Iraq at 46.74.

**Table 3.** Countries with the Ten Highest Number of Attacks and Corresponding Total Fatalities and Injuries

Country	Number of Attacks (N = 565)	Total Fatalities (N = 980)	Total Injuries (N = 7649)	Fatalities per Attack	Injuries per Attack
United States	118 (20.9%)	11 (1.1%)	1148 (15.0%)	0.09	9.73
Russia	49 (8.7%)	52 (5.3%)	202 (2.6%)	1.06	4.12
Iraq	43 (7.6%)	157 (16.0%)	2010 (26.3%)	3.65	46.74
Japan	40 (7.1%)	41 (4.2%)	1345 (17.6%)	1.03	33.63
United Kingdom	30 (5.3%)	0 (0.0%)	3 (0.04%)	0	0.10
Afghanistan	25 (4.4%)	33 (3.4%)	988 (12.9%)	1.32	39.52
China	24 (4.3%)	1 (0.1%)	154 (2.0%)	0.04	6.42
Israel	22 (3.9%)	12 (1.2%)	180 (2.4%)	0.55	8.18
Cambodia	19 (3.4%)	228 (23.3%)	507 (6.6%)	12.00	26.68
India	16 (2.8%)	2 (0.2%)	75 (1.0%)	0.13	4.69
**Subtotal**	**386 (68.3%)**	**537 (54.8%)**	**6612 (86.4%)**	**1.39**	**17.13**
Other Countries (n = 55)	179 (31.7%)	443 (45.2%)	1037 (13.6%)	0.40	5.79

## Discussion

While there is no universal definition of VNSAs, most academics agree with the basic concept that VNSA often refers to any individual, group of individuals, or organization willing and capable of engaging in illicit acts and unsanctioned violence to achieve their goals. Such VNSAs may include insurgencies, terrorist organizations, drug trafficking cartels, transnational gangs, paramilitary groups, and corporations such as private military contractors. They neither directly nor officially represent a recognized state, but they may be supported by state actors.^11^

The current analysis of the VNSA Event database has given insight into a topic that may be even more relevant in the future, with conflicts falling into the grey zone between war and peace, and within the paradigm of hybrid warfare. Hybrid warfare, a topic much discussed under the Counter-Terrorism Medicine (CTM) framework, is often described as a mix of conventional warfare, irregular warfare, terrorism, criminality, and different types of other hybrid threats such as CBRN and cyberattacks.^12,13^ Traditionally, the use of CBRN weapons has been seen as an irrational high-risk act for VNSAs and state players. However, in the past few decades, the use of CBRN weapons in targeting civilian settings and to assassinate political targets indicates a dangerous erosion in the honor of international conventions.^14,15^

### Chemical Agents

Chemical agents are of particular concern when it comes to weapons selection by VNSAs. They are by far the most commonly used CBRN weapons, involved in 434 of 565 of all VNSA CBRN events. They are also the most deadly and injurious, with an average of 2.22 fatalities and 17.37 injuries per attack.

The education required to acquire, manufacture, and deploy simple chemical weapons may also be significantly less than other CBRN weapons, often requiring no more than a college or Masters-level knowledge of chemistry, as demonstrated in the 1995 Tokyo subway attack, which was spearheaded by Masami Tsuchiya, who had a Master’s degree in organic chemistry.^16^ Also, VNSAs need not go to the trouble of manufacturing agents at all, as powerful pharmaceuticals such as fentanyl, remifentanil, carfentanyl, or halothane are readily available on the dark web, and commonly used industrial chemical agents such as chlorine gas can be deployed for nefarious purposes.^3,17,18^

The use of drones by terrorist groups is well-known; drones are used to gather intelligence on secure areas using visual, thermal, and infrared technology, thereby circumventing conventional defenses. Modifying the drone to carry a small payload (chemical, biological, or radiological) and a specialized dissemination device is possible and plausible. In January 2017, the Islamic State of Iraq and Syria (ISIS) started using commercial drones to provide reconnaissance and targeting information against coalition forces and began showing interest in conducting drone-based chemical or biological weapon attacks.^19^ This level of sophistication certainly raises concerns about the possibility of the deployment of these agents in a coordinated or complex multi-modal attack.^4^

Beyond the direct use of chemical weapons, the potential release of toxic chemicals after an attack on chemical manufacturing or storage facilities has been recognized as a serious threat by the United States Cybersecurity and Infrastructure Security Agency (Arlington, Virginia USA) and is addressed through the Chemical Facility Anti-Terrorism Standards.^20^ In addition to the direct health effects, environmental, economic, and long-term health effects of toxic or carcinogenic substances can be felt for years.^21^

The likelihood of emerging chemicals is a new reality. Researchers have reported the use of AI generative technology, using open-access databases, to create novel molecules predicted to be more toxic than known chemical warfare agents in less than six hours. It is therefore entirely possible that these agents can circumvent national and international lists of watched or controlled precursor chemicals.^22^

### Biological Agents

In consideration of biological agents, unlike biological warfare where the goal is the intentional use of a modified biological agent to cause massive loss of human life, bioterrorism is better defined as a method chiefly designed to disrupt a way of life and make a population acutely aware of their vulnerability.^23^ Not discounting the loss of lives or the prolonged severe illness experienced by some of the victims of inhalational anthrax after the September 11, 2001 terrorist attack in the United States, the economic, social, and political disruption resulting from this covert act of bioterrorism were of enormous magnitude. Thirteen years after the September 11 attack, the federal budget for biodefense was nearly 11-times greater than it was at the time of the anthrax attacks.^24^

Globally, there are well over 50 high-containment Biosafety Level (BSL)-4 laboratories either in operation or under construction spread throughout Asia, Africa, Europe, Russia, and the United States. These labs carry out some of the most dangerous manipulations of pathogens with pandemic potential. With each experiment comes opportunities for accidental exposures or deliberate acts of sabotage or theft that could lead to release and dissemination.^25^

Historical accounts of catapulting infected bodies over ramparts were some of the earliest accounts of biological warfare, and the deliberate release of biological agents in warfare accounts for the deaths of thousands over the past hundred years. For example, during the Second World War, the Japanese army poisoned more than 1,000 water wells in Chinese villages to study cholera and typhus outbreaks.^26^ With advances in biotechnologies and the commercialization of home Clustered Regularly Interspaced Short Palindromic Repeats (CRISPR) kits, the plausibility of home-made bioweapons is becoming more likely.^5^ In addition, with drones and other technologies such as nanorobots and biologically modified insect vectors, the dispersion of biological agents becomes more possible, potentially deadlier, and “limitless.”^27^ The combination of genomic technologies with AI, machine learning, automation, affective computing, and robotics will potentially increase the lethality of “old world biologicals” while creating newer and more deadly agents.^25^

### Radiation and Nuclear

Much has been written and discussed over the past several decades on the risks of nuclear theft and terrorism.^7^ From lost nuclear weapons to basement “nukes,” the dangers and global repercussions of an intentional nuclear event are clear, and nuclear security remains a top global priority.^28,29^ The recent standoff between Russia and Ukraine over the Zaporizhzhia nuclear power plant and the 2020 cyberattacks on United States’ nuclear weapons agencies have re-ignited discussions around the protection of critical infrastructure and the risks of both unintentional and intentional damage to such facilities, resulting in a radiological disaster.^8,30–32^

Reports of black-market radioactive components for “dirty bombs” remains an on-going concern and a much more plausible scenario. As an example, Belgian investigators in 2016 discovered terrorists monitoring an employee at a highly enriched uranium reactor that produces medical isotopes for a large part of Europe.^9,33^ Cesium-137 used in blood irradiators and other medical devices also represents a concern given the ease of dispersibility in its powder form, with many experts calling for the elimination of the use of such high-activity radiation sources due to security concerns.^34^

## Limitations

The VNSA CBRN Event database aims to be a comprehensive record of global events but may potentially miss events that are not readily available on publicly accessible sources. Specifically, the database may disproportionately represent events from countries with more established news sources and fail to represent events from communities with reporting restrictions, such as undeveloped media systems or non-transparent government policies. In addition, databases are inherently susceptible to data entry errors or missing data, although this study methodology and data extraction process demonstrates few missing data for the variables of interest.

Event agent and weapon detail coded according to the intended agent/weapon instead of the eventually acquired or used agent/weapon may limit the usefulness of this study in preventing and mitigating eventual attacks. For example, regulatory bans may be misdirected toward intended substances instead of eventual substances. However, there is likely inherent usefulness in identifying intended agents or weapons to implement preventive actions, especially when intended agents are more available to or operationalizable by non-state threat actors.

## Conclusion

Violent Non-State Actors were responsible for 565 unique events around the world involving the use of CBRN weapons from 1990-2020. This resulted in 980 fatalities and 7,649 injuries; with chemical weapons used in 434 events resulting in 965 fatalities and 7,540 injuries, biological weapons in 123 events with 19 fatalities and 59 injuries, radiological weapons in 57 events with zero fatalities and 50 injuries, and nuclear weapons in 18 events with zero fatalities and zero injuries. The United States (118), Russia (49), and Iraq (43) accounted for the top three countries where these events occurred. While CBRN events remain relatively rare, technological advances have the potential to facilitate the use of such weapons as part of a hybrid warfare strategy with significant repercussions for civilian health and health care systems.
